# Epilepsies et crises épileptiques aiguës chez l'enfant en Afrique subsaharienne: défis et espoirs

**DOI:** 10.11604/pamj.2016.23.58.3273

**Published:** 2016-02-29

**Authors:** Célestin Kaputu-Kalala-Malu

**Affiliations:** 1 Service of Child Neurology, Department of Neurology, Kinshasa School of Medicine, University of Kinshasa, Republic Democratic of Congo

**Keywords:** Epilepsie, enfant, Afrique Subsaharienne, Epilepsy, children, sub-Saharan Africa

## Abstract

L’épilepsie est une maladie neurologique fréquemment rencontrée en Afrique Subsaharienne (ASS) au sein de la population pédiatrique. Bien que sa prévalence exacte au sein de cette population ne serait pas connue, il y a lieu de penser qu'elle n'est pas très différente de celle de la population générale à cause de l’âge précoce de son début - avant 20 ans dans plus 60% des cas - et à l'absence de la distribution bimodale retrouvée dans les pays industrialisés. Tout au long de cette revue de la littérature, une réalité se dégage: la prise en charge globale de l'enfant épileptique en ASS est inadéquate. Pour inverser cette tendance, les défis à relever semblent être de trois ordres. Outre la recherche, ces défis comportent une dimension thérapeutique et une dimension éducationnelle. Il y a nécessité de former des médecins à la pratique de la neurologie pédiatrique tout en créant des conditions de travail qui limitent la fuite des cerveaux. Il est urgent d'identifier et de combler les besoins nécessaires à la prise en charge correcte des enfants épileptiques selon les pays et les régions (indisponibilité des médicaments anti comitiaux, l'absence des guidelines nationaux, le déficit d'encadrement de guérisseurs traditionnels…) enfin, il est impérieux d'encourager la réalisation des études visant à établir l'incidence et la prévalence correctes des épilepsies chez l'enfant afin de faciliter la pose des jalons des mesures de lutte ciblées.

## Abstract (en)

Epilepsy is a neurological disease common among children in sub-Saharan Africa (SSA). Although its exact prevalence in this population is unknown, it is probably similar to that found in the general population because of its early onset (60% starts before the age of 20 years) and the absence of bimodal distribution found in western countries. This review acknowledges the reality that current management of pediatric epilepsy in SSA is imperfect. To reverse this trend, the challenges are three fold: better education, improved therapeutics and more research. Doctors need to be trained in the practice of pediatric neurology and provided with working conditions that limit the brain drain. The resources necessary for the proper management of children with epilepsy in SSA need to be identified and provided (better availability of antiepileptic drugs, clear national guidelines, proper registration of traditional healers etc…). Finally, it is imperative that research be conducted to establish accurate incidence and prevalence figures for pediatric epilepsy, so that targeted control measures can be implemented.

## Introduction

La partie de l'Afrique localisée au sud du désert de Sahara est considérée comme une région distincte de celle des six pays formant la pointe septentrionale de l'Afrique, l'Algérie, l'Egypte, la Libye, le Maroc, la Tunisie et le Sahara occidental [1]. L'Afrique subsaharienne (ASS) est une région très variée, qu'il s'agisse de la population, des niveaux de revenu, ou de la composition de la production. Des taux élevés de croissance démographique et des économies à taux de croissance faible ou stagnant dans la majeure partie de la région y compromettent les efforts de modernisation et de développement; quoique, au cours de la décennie écoulée, la plupart des pays d'Afrique subsaharienne aient enregistré durablement des taux de croissance qui leur semblaient auparavant hors de portée, quelques-uns de ces états restent « fragiles » selon la classification du fond monétaire international. Leur évolution économique peut être fortement influencée par des événements non économiques, notamment des conflits civils ou des périodes de reprise au lendemain de troubles civils [2].

L'ASS compterait plus d'un milliard d'habitants, dont plus de la moitié sont âgés de moins de 15 ans. Le tiers de la population vit dans des zones urbaines avec des conditions sanitaires défavorables. En 2002, l'espérance de vie était de 45,8 ans. Elle enregistre 23% des naissances mondiales et 42% des décès d'enfants dans le monde. La mortalité des enfants de moins de 5 ans était de 164,2 pour 1 000 enfants. Seulement 46,4% de la population rurale a accès à l'eau potable et 55% a accès à des équipements hygiéniques. La présence de facteurs environnementaux tropicaux endémiques (vectoriels, toxiques, infectieux, etc…) y joue aussi un grand rôle dans la progression des maladies. Le déficit en ressources humaines y constitue une grande préoccupation: elle compte seulement 1,3% des travailleurs de la santé alors qu'elle porte à elle seule 25% de la charge mondiale de maladies [3].

Les dépenses de santé sont réduites, au détriment de la qualité et de l'accès aux soins. Les travaux de recherches médicales restent limités par manque de moyens. L'information demeure insuffisante dans le domaine de la recherche médicale en Afrique. A titre d'exemple, plus de 6 millions d'articles sur la recherche médicale sont apparus dans le monde entre 1996 et 2006 dont seulement 55 000 articles concernaient l'Afrique. Quant à la neurologie pédiatrique, elle est encore méconnue et inexistante dans plusieurs pays d'ASS. En 2010, on comptait seulement 6 neuropédiatres dans toute l'Afrique de l'Est dont 5 au Kenya. Ceci pourrait expliquer la relative pauvreté des études consacrées à l’épilepsie infantile. Il est donc difficile d'analyser l'incidence et la prévalence réelles de l’épilepsie infantile à l’échelle des sous régions et même des pays [4–6].

## Méthodes

Il s'agit d'une revue de la littérature. Les données ont été rassemblées grâce aux recherches effectuées via Pubmed et Google Scholar essentiellement. Cette recherche s'est focalisée sur l’épidémiologie des épilepsies chez l'enfant en Afrique subsaharienne, la description des crises épileptiques aiguës ainsi que leur prise en charge. Les facteurs étiologiques et/ou de risque ainsi que les aspects thérapeutiques et socio-culturel font également l'objet d'une analyse approfondie.

## Résultats

### Incidence et prévalence de l’épilepsie chez l'enfant en Afrique subsaharienne

L'Organisation Mondiale de Santé estime qu'environ 50 millions d’êtres humains souffriraient de l’épilepsie dont 80% vivraient dans les pays en voie de développement, [7]. L'incidence exacte pas plus que la prévalence de l’épilepsie au sein de la population pédiatrique ne sont toutefois pas connues dans la région du monde qui nous concerne au sud du Sahara [8]. Les études consacrées à ce phénomène étant fragmentaires [9]. La stigmatisation de la maladie, le manque criant du personnel qualifié, particulièrement de neuropédiatres, la quasi absence des appareils EEG et la modicité des sommes allouées à la recherche expliquent ce manque de données. D'une manière générale et sans considération d’âge, l'incidence de l’épilepsie en ASS est estimée à 63-158 / 100.000 habitants / an contre 40-70 dans les pays industrialisés. La prévalence quant à elle, n'est pas aussi élevée qu'on ne saurait s'y attendre. Elle est néanmoins estimée 2 à 3 fois supérieure par rapport à celle enregistrée dans les pays industrialisés. Elle varie entre 7 et 14,8 ‰ selon une récente étude menée dans 5 pays différents lorsque seulement l’épilepsie active est prise en compte [10]. De manière générale, elle varie entre 5,2% et 74% selon les régions et méthodes utilisées [5, 11]. Les données pédiatriques sont fragmentaires. Dans une étude faite à Bamako (Mali) sur une population âgée de 3 à 15 ans, il a été estimé une prévalence de 11,3‰ [12]. Dans une étude sénégalaise réalisée en milieu scolaire chez les enfants âgés de moins de 10 ans [13], la prévalence a été estimée à 21‰. Les données plus ou moins récentes viennent du Kenya où l'incidence de l’épilepsie chez les sujet de 6 à 9 ans a été estimée à 187/ 100.000 habitant/ an [9]. Dans cette même étude, la prévalence totale de l’épilepsie (active et non active) a été calculée à 41‰ et celle de l’épilepsie active à 11‰. (95% CI: 5-15). Les chiffres globaux sur l'incidence et la prévalence de l’épilepsie en ASS sont donc transposables sur la population pédiatrique. Ceci est d'autant plus compréhensible que celle-ci commence avant 20 ans dans plus de 60% de cas [5, 11]. Il faut rappeler que la majorité - plus de 50% - de la population en Afrique Subsaharienne est âgée de moins de 15 ans [3, 6].

### Incidence des crises épileptiques aiguës chez l'enfant en Afrique subsaharienne

Il n'est pas facile de faire la distinction entre épilepsie et crises épileptiques récurrentes survenant dans un contexte fébrile chez l'enfant en ASS compte tenu de la modicité de moyens d'investigation. De manière générale, une crise survenant dans un contexte de fièvre peut être symptomatique (d'une méningite par exemple), faire évoquer le diagnostic d'une convulsion fébrile, n’être qu'une crise initiale chez un enfant épileptique ou faire partie d'un syndrome épileptique spécifique (exemple du syndrome de Dravet) [14–16]. Au sud du Sahara, les crises épileptiques aiguës sont fréquentes. Elles constituent jusqu’à 18,3% des consultations aux urgences pédiatriques. Elles sont associées aux infections, donc possiblement symptomatiques, dans 80% des cas. Leur incidence serait de 423 /100000 habitants/an lorsque l'on considère les enfants âgés de 0 à 13 ans et de 879 chez les moins de 5 ans [17]. Quant à l’état de mal épileptique, son incidence serait de 35 / 100000/ an chez les enfants âgés de 1 à 13 ans, de 52 chez ceux âgés de 1 à 11 mois et de 85 chez ceux âgés de 12 à 59 mois [18]. Ces chiffres constituent au moins le double de ce qui est décrit chez l'enfant âgé de moins de 16 ans à Londres où elle serait de 18-20 / 100000/an [19].

### Pronostic

Pour des raisons déjà évoquées ci-dessus, il n'est pas aisé de trouver, dans la littérature africaine, les données en rapport avec l’évolution à long terme de l’épilepsie dans la population pédiatrique. Nous allons donc piocher dans les études disponibles afin de déceler celles qui ont réservé quelques lignes à la population pédiatrique. Tekle-Haimanotet al. [20] estimaient le taux brut de mortalité annuel chez les sujets épileptiques à 31,6%, soit 2 fois plus que le taux de mortalité observé dans la population générale (16,4%). La plupart des décès observés dans cette étude étaient directement liés à l’état de mal épileptique. L’étude réalisée en Tanzanie par Jilek-Aall et Rwiza [21] confirmait les résultats de Tekle-Haimanotet al: les taux de survie actuarielle de 164 épileptiques traités avec un anticomitial de fond étaient environ 2 fois plus faibles que ceux d'une population générale à âge équivalent. Plus de la moitié des décès était liée aux crises ou à leurs conséquences. Dans cette étude, parmi les 164 patients épileptiques revisités 30 ans après le début de la thérapeutique anticomitiale, 52,4% n'avaient plus de convulsions, 36% avaient connu la réduction de leur crises, 7,9% n'avaient connu aucun changement et 0,6% avaient vu leurs crises s'aggraver. Dans une étude indirecte et rétrospective effectuée au Malawi [22], Watts a noté que de nombreux patients entraient en rémission spontanée avec ou sans un traitement anticomitial de fond. Quand bien même ces études n'aient pas visé spécifiquement les populations pédiatriques, il est important de noter que dans les deux dernières, près de 80% des patients n'avaient pas encore 20 ans au moment du diagnostic. Pour ce qui regarde l’évolution au décours des crises aiguës, la mortalité au sein de la population pédiatrique admise dans un hôpital rural au Kenya était de 3,1%. Douze pourcent des patients avaient présenté des séquelles neurologiques sévères à la sortie. Cette mortalité hospitalière grimpait à 15% en cas d’état de mal épileptique. À part l’état de mal lui-même, la mortalité était associée au caractère focal de crises, au coma, à l'acidose métabolique, à la méningite bactérienne et à la bactériémie [17, 18]. Pour ce qui touche aux crises associées à la fièvre, la mortalité (0,4%), semblait être liée au caractère répétitif des crises à l'analyse multivariée dans une étude effectuée dans 2 services pédiatriques à Kinshasa, République démocratique du Congo [23]. Il est important de relever que le décès survenant au décours d'une crise fébrile reste exceptionnel dans la population européenne. Une étude rétrospective qui a été récemment menée sur la population liégeoise n'a déploré aucun décès [24].

### Types de crises épileptiques

Le diagnostic d'une crise épileptique est d'abord clinique. Un interrogatoire bien conduit auprès des proches peut faire soupçonner une comitialité. Grâce aux nouvelles technologies, ces proches, avisés, peuvent emmener au personnel soignant les images d'une crise épileptique enregistrée à la maison. Mais ces derniers, qui constituent l'unique source d'information, ne connaissent pas assez au sujet de l’épilepsie et sont souvent paniqués lors de la survenue des crises [25, 26]. Le diagnostic peut être affiné grâce à l'EEG et à la perspicacité de celui qui mène l'interrogatoire, mais, le manque des neurologues et des machines d'EEG, la sous-médicalisation, l'intervalle de temps s’écoulant entre le moment du début des crises et celui où le patient est effectivement présenté devant un médecin compètent (ce qui peut contribuer à la modification des crises) sont des facteurs qui peuvent expliquer la sous-estimation de la prévalence des crises partielles et les crises partielles secondairement généralisées au profit de convulsions généralisées tonico-cloniques en ASS. Celles-ci ont en effet plus de chance d’être observées et décrites par les proches grâce à leur aspect spectaculaire. Sous réserve de ce qui précède, il existe dans les études une prédominance des crises généralisées tonico-cloniques avec une moyenne de 59% ± 21 [5, 27, 28]. Près de 79% des crises étaient généralisées dans l’étude de Idro. Dans cette même étude, 13,4% des crises étaient focales et 4,2% focales secondairement généralisées. Dans 52,3% des cas, les crises étaient répétitives (2 ou plus). L’état de mal épileptique (défini comme une crise de durée supérieure ou égale à 30 minutes ou la succession de 3 ou plusieurs crises sans recouvrement de la conscience endéans une heure) était survenu dans 10,1% de cas. Les états de mal convulsifs semblent souvent se présenter sous forme focale. Dans une étude portant sur les enfants ayant présenté un état de mal convulsif au Kenya, 39% d'entre eux avaient présenté les crises à début focal [17, 18].

### Etiologies et facteurs de risque

En ASS, la proportion des facteurs de risque ou étiologiques diminue en fonction du nombre d'explorations réalisées. Le médecin ne disposant généralement que des examens complémentaires les plus simples, le diagnostic étiologique est basé principalement sur l'interrogatoire et la clinique. De ce fait, les étiologies varient en fonction de l’âge et de la localisation géographique. Ces facteurs de risque ou étiologiques sont généralement ubiquitaires. Certains, dont les pathologies infectieuses et parasitaires, semblent toutefois spécifiques à l'ASS [5]. Selon une étude multicentrique récente, les causes périnatales (OR 10,23, 95% CI 5,85- 17,88; p=0,001), anténatales (OR 2,15, 95% CI 1,53- 3,02; p=0,001) et les traumatismes crâniens (OR 1,95, 95% CI 1,28- 3,03; p=0,002) sont les plus associées à une forte prévalence d’épilepsie chez enfants âgé de moins de 18 ans [10]. Il semble donc que les épilepsies symptomatiques acquises constituent la grande majorité des cas chez l'enfant au sud du Sahara [29–31]. Une grande proportion de ces épilepsies est attribuable aux causes évitables. Cela va sans dire qu'une réduction de celles-ci devrait découler de l'amélioration des politiques dans le domaine de la santé maternelle et infantile et la généralisation de la lutte contre les maladies infectieuses et parasitaires dont la malaria [32, 33]. Les crises épileptiques aiguës, quant à elles, sont associées aux infections dans plus de 80% des cas tel que corroboré par l’étude de Idro et al portant sur 900 crises aiguës survenues chez les enfants de 3 à 13 ans admis dans un hôpital de district sur une période de 2 ans, au Kenya [17]. Le paludisme dû au plasmodium falciparum était de loin le diagnostic le plus rencontré: 58%. Il était associé au prolongement de la durée des crises, à l'augmentation de la fréquence de crises ainsi qu’à la survenue des états de mal épileptiques (57,1% des états de mal épileptiques étaient dus au paludisme). Une forte parasitémie était particulièrement associée à la fréquence des crises. Les étiologies des crises variaient avec l’âge: le sepsis prédominait en âge néonatal, les méningites pyogènes, les gastroentérites et les infections des voies respiratoires étaient l'apanage des enfants âgés de 2 à 5 mois enfin, le paludisme représentait l'affection la plus importante à partir de 6 mois. Même lors de survenue d'un état de mal convulsif, le paludisme reste la maladie la plus incriminée avec 65% des gouttes épaisses positives et un diagnostic ferme de malaria dans 53% des cas. Suivi de bactériémie (11%) et de méningite: 3% [18]. Le paludisme était encore le diagnostic le plus rencontré (63%) dans une récente étude menée dans deux pays subsahariens (République démocratique du Congo et Rwanda) par Kaputu et al sur une population de 436 enfants âgés de 5 mois à 10 ans présentant des convulsions dont la durée atteignait ou dépassait 5 minutes [34]. Une attention très particulière doit être portée sur les convulsions dites « fébriles ». Elles sont souvent sévères et fréquentes en ASS. Leur fréquence varie selon les études et peut atteindre 67, 3% [35]. Le paludisme est retrouvé à l'origine de ces manifestations [36–39] dans 50 à 68% des cas [5, 11, 40]. Dans les zones d'endémie palustre, la question de savoir si ces crises sont seulement liées à fièvre ou à la souffrance cérébrale directe ou indirecte induite par les infections - à l'instar de la malaria [41] - n'est pas définitivement tranchée. Il faut remarquer que c'est à partir de 6 mois que la malaria devient le principale responsable des crises épileptiques aiguës chez l'enfant en ASS [17]. Or, cet âge correspond à celui à partir duquel on assiste à l'augmentation de la fréquence des convulsions fébriles. Il semble en tous cas prouvé que non seulement la malaria est responsable d'un grand nombre de crises épileptique aiguës complexes (prolongées, répétitives et focales) [10, 42], mais aussi un facteur de risque de survenue d'une épilepsie séquellaire [43, 44].

### Aspects thérapeutique et socio-culturels

Il n'est pas facile de dissocier ces deux aspects tant ils s'influencent mutuellement.

### Importance du gap thérapeutique en Afrique subsaharienne

Selon la ligue internationale de lutte contre l’épilepsie [45], le gap thérapeutique est « la différence entre le nombre des patients souffrant de l’épilepsie active et celui de ceux dont les crises sont correctement traitées dans une population donnée à un moment donné, exprimée en pourcentage. Cette définition inclut le déficit diagnostic et thérapeutique ». Sous ce rapport, la prise en charge de l’épilepsie chez l'enfant en ASS est en inadéquation avec son incidence et sa prévalence. Le gap thérapeutique varie entre 23% et 100% selon les régions [46–49]. Les récentes données de la littérature révèlent qu'en ASS, rares sont des pays qui possèdent un protocole national de prise en charge de l’épilepsie et pire, de la crise épileptique prolongée de l'enfant [50]. En outre, l'ASS n'a toujours pas un accès facile ni aux antiépileptiques le plus récents ni aux autres possibilités thérapeutiques déjà largement utilisées dans les pays dits « développés » (chirurgie de l’épilepsie, régime cétogène…) [51]. Ce gap thérapeutique serait lié au manque criant de personnel qualifié, au prix élevés des médicaments et/ou à leur indisponibilité ainsi qu'aux croyances culturelles mystico-religieuses qui entourent encore cette maladie [49, 52].

### Efficacité des anticonvulsivants utilisés en première intention dans la prise en charge de crises convulsives aiguës prolongées en Afrique subsaharienne

Devant une crise épileptique qui risque de se prolonger, outre la recherche et le traitement de la cause sous-jacente, l'administration d'un médicament sûr, efficace, d'administration facile et possédant une longue durée d'action reste le fer de lance de la prise en charge pour le personnel de santé de première ligne. Généralement, l'administration précoce d'un médicament GABAergique (benzodiazépine) permet le plus souvent d’éviter la prolongation de la crise pour peu que ceux qui assistent à la crise connaissent non seulement l'existence mais aussi le contenu du protocole de prise en charge. Kaputu et al, ont malheureusement démontré que le personnel soignant - tant médical qu'infirmier - ignore le plus souvent l'attitude à adopter dans pareil cas [53]. Jusqu’à il y a peu, seulement 7 pays possédaient un protocole national de prise en charge de l’état de mal épileptique de l'enfant en ASS. Dans 23 pays visités, le diazépam (DZP) était le seul anticonvulsivant de première ligne. En République démocratique du Congo, un pays qui compte près de dixième de la population d'ASS, il n'y a pas d'autres options au-delà du diazépam [50]. Il va donc sans dire que le DZP est le seul anticonvulsivant dont on doit évaluer l'efficacité dans la prise en charge de crises épileptiques aiguës prolongées en ASS. Il constitue d'ailleurs souvent la seule drogue de référence avec laquelle quelques comparaisons cliniques ont été faites. Administré en solution rectale chez 55 enfants nigérians, le diazépam était à même de stopper les crises dans 71% de cas endéans 5 minutes. Les crises qui avaient duré moins de 15 minutes avant l'administration du diazépam en solution intrarectale étaient plus sensibles (81%) par rapport à celles qui avaient duré plus de 15 minutes (46%) [54]. Administré par voie intraveineuse (IV) (0,3mg / kg) ou intrarectale (IR) (0,5 mg / kg), le DZP n'a pas empêché la récurrence des convulsions chez la moitié des enfants avec malaria admis dans l’étude de Oguto et al [55], obligeant le personnel conduisant l’étude à recourir aux anticonvulsivants à longue durée d'action. Ce qui est important de souligner est que certains enfants ayant été traité avec du DZP/ IR ont eu leur récidive alors que la concentration plasmatique du DZP se trouvait dans la fourchette généralement admise pour une activité anticonvulsivante optimale (entre 200 et 600 ng/ ml). Ce qui a poussé les auteurs à conclure que l'effet prophylactique du DZP est faible. Dans cette même étude, le DZP / IR n'a pas arrêté les convulsions dans le tiers des cas. L'on a aussi noté une plus grande variabilité des concentrations plasmatiques du DZP au sein du groupe DZP / IR que le dans le groupe DZP / IV [55]. Chez les enfants souffrant de la malaria, le caractère focal de la convulsion, la malaria cérébrale (parasitémie positive, le score de Blantyre < 2 chez un enfant incapable de localiser un stimuli douloureux plus d'une heure après le contrôle de crise sans évidence d'infection du système nerveux central ni d'hypoglycémie) et l'hyperglycémie sont des facteurs associés à l’échec thérapeutique après administration du DZP/ IR (0,5mg/kg) ou du Midazolam (MDM) buccal (0,5mg/kg). Chez ces enfants impaludés, les crises focales, les crises multiples et la malaria cérébrale étaient associés de manière indépendante au risque de récidive. Dans les deux groupes, les facteurs d’échec thérapeutique étaient le caractère focal et la malaria cérébrale. Les convulsions prolongées étaient plus susceptibles de ne pas répondre à l'administration d'un anticonvulsivant dans le groupe DZP par rapport au groupe MZM. Le risque de récidive était beaucoup plus important dans le groupe DZP par rapport au groupe MZM [56]. Lorsque les deux traitements étaient appliqués chez les enfants qui convulsaient sans considération d’étiologie sous-jacente, 43% des convulsions n'ont pu être stoppées 10 minutes après administration du DZP intrarectal contre 30,3% après MZM buccal [57]. A part le DZP et le MZM, le lorazépam (LZP) et paraldéhyde ont étaient aussi objets d'essais cliniques en ASS. Chez les enfants présentant la malaria sévère, une dose (0,1 mg/kg) du LZP administré par intraveineuse (IV) ou intramusculaire (IM) avait stoppé toutes les convulsions endéans 16 et 27 minutes respectivement. Septante-deux heures après, la même dose était capable de prévenir la récidive dans 73% et 91% des cas après l'administration du LZP en IV et en IM respectivement [58]. Administré par voie intranasale, le LZP (100µg/kg) avait stoppé 73% des convulsions endéans 10 minutes contre 61,3% après l'administration de paraldéhyde (0,2ml/kg) en IM dans l’étude de Ahmad et al [59]. Plus récemment, Kaputu et al ont comparé le DZP IR (0,5mg /kg) au LZP sublingual / buccal (0,1 mg/kg) chez l'enfant simultanément au Rwanda et en République démocratique du Congo [60]. Selon cette étude, le LZP sublingual / buccal à la dose de 0,1 mg/kg était moins efficace par rapport au DZP (56% des crises stoppées endéans 10 minutes contre 76%). Visiblement, ces essais thérapeutiques n'ont pas encore réussi à ébranler l'usage presque exclusif du DZP malgré un besoin pressant de trouver un médicament sûr, efficace, d'administration facile mais possédant une longue durée d'action pour gérer les convulsions longues, répétitives, focales et récurrentes caractérisant la malaria cérébrale en ASS.

### Aspects socio-culturels

Le gap thérapeutique qui caractérise l'ASS est l'une des conséquences des croyances farfelues au sujet de l’étiologie et de la prise en charge de l’épilepsie. Dans presque toutes les sociétés en ASS et à des degrés divers, l’épilepsie est très stigmatisant car, considérée comme d'origine mystérieuse. Ceci est corroboré par une étude faite au Cameroun où 30,2% des personnes interrogées pensaient qu'elle ne peut être guérie que par les guérisseurs traditionnels [61]. Dans cette même étude, 3,9% des personnes interrogées trouvaient qu'il ne servait à rien de proposer un traitement aux épileptiques soulignant par là son caractère incurable. Soixante-huit pourcent d'interviewés ne pouvaient épouser un épileptique. Les épileptiques passent donc pour des véritables parias de la société à cause de la contagiosité présumée de leur mal. Même dans la population des élèves du niveau secondaire du Cameroun, 58% étaient convaincus de sa contagiosité [62]. En ASS, l’épilepsie a un impact délétère sur la scolarité et le devenir professionnel des enfants qui en souffrent. Au Nigeria, près de 20% d'enfant quittent l’école à cause de leur épilepsie et 39,5% de ceux qui atteignent le niveau universitaire accusent des contre-performances par rapport à leurs condisciples non-épileptiques. Ceci engendre les difficultés de socialisation des malades (étude, mariage, contacts humains…) qui, vite, sédimentent dans les couches socioprofessionnelles les plus basses [5, 52, 63].

## Conclusion

Les récentes recommandations de l’« International Child Neurology Association » et de l’ « African Child Neurology Association » pourraient aider à poser les jalons d'une prise de conscience de la communauté scientifique subsaharienne visant à améliorer la prise en charge des enfants épileptiques. Ces 2 sociétés savantes ont reconnu la nécessité de former les médecins à la pratique de la neurologie pédiatrique et de créer des conditions de travail qui limitent la fuite des cerveaux. Elles ont suggéré l'identification du rôle que pourraient jouer les guérisseurs traditionnels et la formation de groupes de travail dont l'objectif sera de rendre optimal la prise en charge globale des enfants épileptiques. Elles ont proposé d'identifier et de combler des besoins nécessaires à la prise en charge correcte des enfants épileptiques selon les pays et les régions en ASS (indisponibilité des médicaments anti comitiaux, l’établissement des guidelines nationaux…) et enfin, elles ont encouragé la réalisation des études visant à établir l'incidence et la prévalence correctes de l’épilepsie chez l'enfant afin de faciliter la mise sur pied des mesures de lutte ciblées [8, 64].

### Etat des connaissance sur le sujet

- La prise en charge des crises épileptiques et des épilepsies de l'enfant en ASS est inadéquate
- Les causes les plus souvent évoquées pour expliquer cette situation dramatique sont le manque criant d'un personnel qualifié, le sous-équipement
- Enfin, le fait que la pratique de la neuropédiatrie soit encore à ses balbutiements

### Contribution de notre étude a la connaissance

- Cette revue met en lumière les données épidémiologiques actualisées des épilepsies et crises épileptiques aiguës de l'enfant en ASS
- Elle en décortique les étiologies ainsi que les facteurs de risque avant de réserver une attention particulière aux aspects thérapeutiques
- Un accent particulier a été mis sur la prise en charge des crises épileptiques prolongées de l'enfant africain
- Enfin, cette revue de littérature identifie tout ce qui peut faciliter la pose des jalons des mesures de lutte ciblées
